# Adverse Effects of Simulated Hyper- and Hypo-Phosphatemia on Endothelial Cell Function and Viability

**DOI:** 10.1371/journal.pone.0023268

**Published:** 2011-08-09

**Authors:** Ai Peng, Tianfu Wu, Caihong Zeng, Dinesh Rakheja, Jiankun Zhu, Ting Ye, Jack Hutcheson, Nosratola D. Vaziri, Zhihong Liu, Chandra Mohan, Xin J. Zhou

**Affiliations:** 1 Department of Nephrology, Shanghai Tenth People's Hospital of Tongji University, Shanghai, China; 2 Department of Pathology, UT Southwestern Medical Center, Dallas, Texas, United States of America; 3 Division of Rheumatology, Department of Internal Medicine, UT Southwestern Medical Center, Dallas, Texas, United States of America; 4 Research Institute of Nephrology, Jinling Hospital, Nanjing University School of Medicine, Nanjing, China; 5 Division of Nephrology and Hypertension, University of California Irvine, Irvine, California, United States of America; L' Istituto di Biomedicina ed Immunologia Molecolare, Consiglio Nazionale delle Ricerche, Italy

## Abstract

**Background:**

Dysregulaiton of phosphate homeostasis as occurs in chronic kidney disease is associated with cardiovascular complications. It has been suggested that both hyperphosphatemia and hypophosphatemia can cause cardiovascular disease. The molecular mechanisms by which high or low serum phosphate levels adversely affect cardiovascular function are poorly understood. The purpose of this study was to explore the mechanisms of endothelial dysfunction in the presence of non-physiologic phosphate levels.

**Methodology/Principal Findings:**

We studied the effects of simulated hyper- and hypophosphatemia in human umbilical vein endothelial cells *in vitro*. We found both simulated hyperphosphatemia and hypophosphatemia decrease eNOS expression and NO production. This was associated with reduced intracellular calcium, increased protein kinase C β2 (PKCβ2), reduced cell viability, and increased apoptosis. While simulated hyperphosphatemia was associated with decreased Akt/p-Akt, Bcl-xl/Bax ratios, NFkB-p65 and p-Erk abundance, simulated hypophosphatemia was associated with increased Akt/p-Akt and Bcl-xl/Bax ratios and p-Mek, p38, and p-p38 abundance.

**Conclusions/Significance:**

This is the first demonstration of endothelial dysfunction with hypophosphatemia. Our data suggests that both hyperphosphatemia and hypophosphatemia decrease eNOS activity via reduced intracellular calcium and increased PKCβ2. Hyperphosphatemia also appears to reduce *eNOS* transcription via reduced signaling through PI3K/Akt/NF-kB and MAPK/NF-kB pathways. On the other hand, hypophosphatemia appears to activate these pathways. Our data provides the basis for further studies to elucidate the relationship between altered phosphate homeostasis and cardiovascular disease. As a corollary, our data suggests that the level of phosphate in the culture media, if not in the physiologic range, may inadvertently affect experimental results.

## Introduction

Phosphate is an essential mineral that is a necessary component of DNA and RNA, is essential for cellular metabolism as an energy source in the form of ATP, and is critical for proper bone development. Serum phosphate levels are regulated by an interplay of dietary intake, parathormone (PTH), 1,25-dihydroxyvitamin D, and fibroblast growth factor 23 (FGF23) that act on the intestine, skeleton, and kidneys [1]. Of these, the kidney is the major site for minute-to-minute regulation of phosphate homeostasis; approximately 70% of the filtered phosphate is reabsorbed within the proximal tubule where the sodium-phosphate co-transporters Npt2a and Npt2c are expressed. PTH reduces the expression of Npt2a and Npt2c in the apical membrane of the proximal tubule [1]. High PTH levels, as in hyperparathyroidism, lead to renal phosphate wasting and hypophosphatemia, while low PTH levels, as in hypoparathyroidism, lead to increased renal phosphate reabsoption and hyperphosphatemia. Similar to PTH, FGF23 suppresses phosphate reabsorption in the proximal tubule. However, PTH and FGF23 have opposite effects on 1,25-dihydroxyvitamin D production. PTH increases and FGF23 decreases the proximal renal tubular expression of 25-hydroxyvitamin D 1α-hydroxylase that catalyzes the conversion of 25-hydroxyvitamin D to 1,25-dihydroxyvitamin D. The latter in turn regulates serum phosphate concentration by increasing intestinal calcium and phosphate absorption [1].

Chronic kidney disease (CKD) is associated with accelerated atherosclerosis, hypertension and increased incidence of death from myocardial infarction, stroke, and heart failure [2]. Several factors contribute to the pathogenesis of CKD-induced atherosclerosis and cardiovascular disease; these include oxidative stress, inflammation, dyslipidemia and hypertension [3], [4], [5], [6]. In addition, dysregulation of phosphate homeostasis, a common feature of CKD, can contribute to the cardiovascular complications. In an earlier study Tonelli et al [7] found a graded independent relation between higher levels of serum phosphate and the risk of death and cardiovascular events among people with prior myocardial infarction, most of whom had serum phosphate levels within the normal range. They further showed that elevated serum phosphate levels were associated with increased risk of new-onset heart failure, myocardial infarction, and the composite of coronary death or nonfatal myocardial infarction [7]. Hyperphosphatemia has been shown to induce acute endothelial dysfunction *in vivo* and exposure to a phosphorus load has been shown to increase reactive oxygen species production, induce apoptosis, and decrease nitric oxide (NO) production in endothelial cells *in vitro* [8], [9]. The decreased NO production may occur because of inactivation of endothelial nitric oxide synthase (eNOS) caused by phosphorylation at Thr497 via activation of protein kinase C (PKC) by phosphate. In a double-blind crossover study, flow-mediated brachial artery dilation was measured before and two hours after meals containing 400 mg or 1200 mg of phosphorus. The higher dietary phosphorus load increased serum phosphate at two hours and significantly reduced flow-mediated brachial artery dilation indicating a causal relation between endothelial dysfunction and acute postprandial hyperphosphatemia [10]. On the other hand, hypophosphatemia can also cause cardiovascular disease including heart failure after cardiac surgery and cardiac arrest in patients undergoing treatment for diabetic ketoacidosis with hypertriglyceridemia [11], [12]. Hypertension and metabolic syndrome are also associated with hypophosphatemia and increased risk of cardiovascular disease [13]. Hypophosphatemia may lead to a decreased intracellular inorganic phosphate and mitochrondrial dysfunction leading to decreased ATP synthesis [13].

Endothelial dysfunction is a common and critical step in the development of cardiovascular diseases [14], [15]. In endothelial cells, NO is produced from L-arginine and molecular oxygen by the constitutively expressed eNOS. NO is a potent vasodilator and a key determinant of cardiovascular homeostasis by virtue of its role in regulation of arterial blood pressure, vascular remodeling, and angiogenesis as well as its anti-inflammatory and anti-thrombotic actions [16]. Endothelial dysfunction is characterized by reduced eNOS activity and/or expression and decreased NO availability, which is typical of patients with cardiovascular disease [17]. The activity of eNOS is regulated by multiple mechanisms that include transcriptional and epigenetic regulation of mRNA, and post-translational regulation of the protein by reversible palmitoylation and caveolar targeting, intracellular calcium levels and calmodulin binding, reversible phosphorylation of serine and threonine residues, and reversible S-nitrosylation [18], [19], [20]. Of note, phosphorylation at Ser-1177, Ser-635, and Ser-617 are stimulatory, while phosphorylation at Thr-495 and Ser-116 are inhibitory [20]. The stimulatory phosphorylation of eNOS residues Ser-1177 and Ser-617 occur in response to flow shear stress that activates phosphatidyl-inositol 3-kinase (PI3K) and its downstream serine/threonine protein kinase, Akt, which in turn phosphorylates eNOS (PI3K-Akt-eNOS pathway) [16], [21], [22]. On the other hand, inhibitory phosphorylation of eNOS at Thr497 occurs via activation of PKC. In endothelial cells, the transcription of eNOS is also regulated by shear stress. Shear stress functions via Src to activate a mitogen-activated protein kinase (MAPK) pathway that includes Ras, Raf/MEK, and ERK1/2. The end-result is the activation of the transcription factor nuclear factor (NF)-kB and initiation of eNOS transcription. Further, signal transduction through the high-density lipoprotein (HDL) and estrogen membrane receptors activates the PI3K-Akt pathway via Src/Ras. While Akt can produce stimulatory phosphorylations of eNOS, it might also activate NF-kB, which in turn can initiate eNOS transcription [23], [24], [25].

The mechanisms by which high or low serum phosphate levels adversely affect cardiovascular function are poorly understood. Since NO and eNOS play a major role in vascular physiology, we studied the effects of low and high phosphate levels on eNOS expression and NO production *in vitro* in human umbilical vein endothelial cells (HUVECs) and also explored the *in vitro* mechanisms that might regulate eNOS expression and NO production.

## Results

### Effect of phosphate concentration on endothelial cell growth, apoptosis, eNOS expression, and NO production

**Figure 1:**
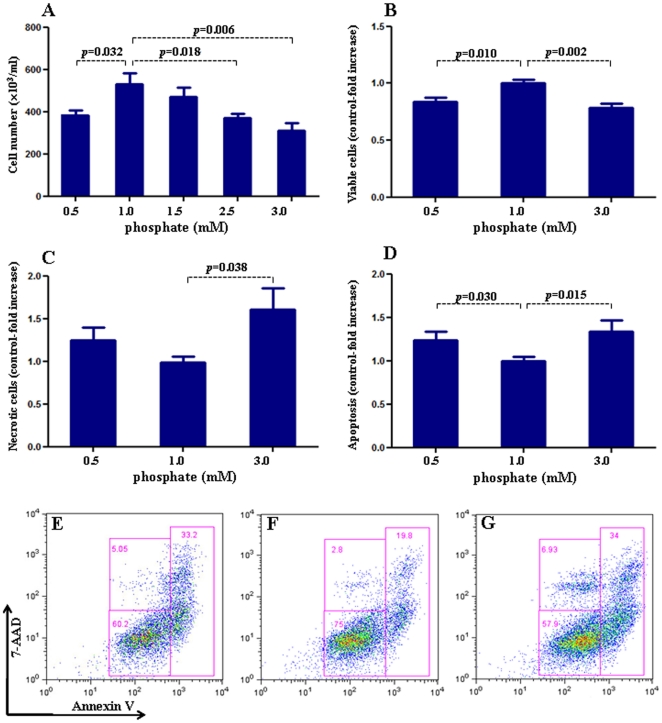
Effect of inorganic phosphate on endothelial cell proliferation and apoptosis. We examined the effect of exposure to different concentrations of inorganic phosphate for 24 hours on human umbilical vein endothelial cell proliferation and apoptosis. In Figure 1A, cell number is determined by light microscopy counting of trypan blue-excluding cells, whereas B–D pertain to flow analysis. Incubation in media simulating hypophosphatemia (0.5 mM) or hyperphosphatemia (3 mM phosphate) resulted in a significant reduction of cell numbers (A), decreased cell viability (B) and increased cell dearth (C, statistically significant only seen in hyperphosphatemia) when compared to cells incubated in the medium containing 1 mM phosphate. Similarly, incubation in media simulating hypophosphatemia (0.5 mM, D & E) or hyperphosphatemia (3 mM phosphate, D & G) resulted in a significant increase in apoptotic cells when compared to cells incubated in the medium containing 1 mM phosphate (D & F). In Figure E–G, data in each quadrant are the percentage of total cells, and shown as mean +/− SEM. p values vs control (1 mM phosphate). N = 6∼9.

**Figure 2:**
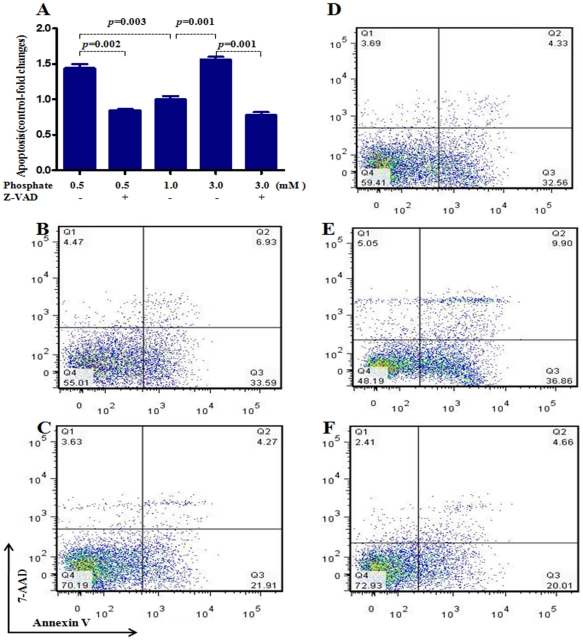
Effect of pan-caspase inhibitor z-VAD in preventing apoptosis caused by simulated hypophosphatemia and hyperphosphatemia. To further define the role of inorganic phosphate on human umbilical vein endothelial cell apoptosis, a pan-caspase inhibitor z-VAD (40 uM) was added to the medium containing various concentrations of phosphate. Incubation with pan-caspase inhibitor z-VAD prevented apoptosis induced by both simulated hyperphosphatemia and hypophosphatemia (Figure 2 A–F). B: hypophosphatemia (0.5 mM); C: hypophosphatemia (0.5 mM) plus z-VAD; D: phosphate (1 mM); E: hyperphosphatemia (3 mM phosphate); F: hyperphosphatemia (3 mM) plus z-VAD. In Figure B–F, data in each quadrant are the percentage of total cells, and shown as mean +/− SEM. p values vs control (1 mM phosphate). N = 6∼9.

**Figure 3:**
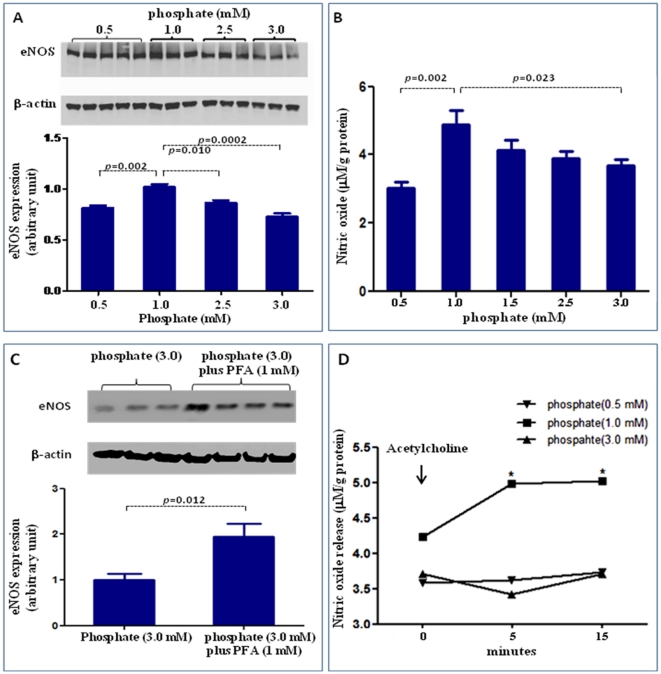
Effect of inorganic phosphate on eNOS expression and NO production . We examined the effect of exposure to different concentrations of inorganic phosphate for 24 hours on NO production and eNOS expression. Incubation in media simulating hypophosphatemia (0.5 mM) or hyperphosphatemia (3 mM phosphate) resulted in a significant reduction in eNOS expression (A) and NO production (B). The down-regulation of eNOS following exposure to simulated hyperphosphatemia (3 mM phosphate) was reversed with co-administration of 1 mM PFA, which is a specific inhibitor of phosphate transport across the cell membrane (C). To determine whether abnormal phosphate levels affected the ability of acetylcholine (a physiological activator of eNOS) to stimulate NO production, a set of cells was incubated with 2 µM acetylcholine and the supernatant NO levels before (0 minute) and 5 minute and 15 minute post acetylcholine stimulation was measured. As shown in (D), incubation in medium with 1 mM phosphate resulted in a significant NO production 5 minute post acetylcholine stimulation and remained elevated at 15 minutes. In contrast, incubation in media simulating both hypophosphatemia (0.5 mM) and hyperphosphatemia (3 mM phosphate) completely prevented acetylcholine-induced stimulation of NO generation, pointing to endothelial dysfunction. p values vs control (1 mM phosphate). N = 6∼10.

**Figure 4:**
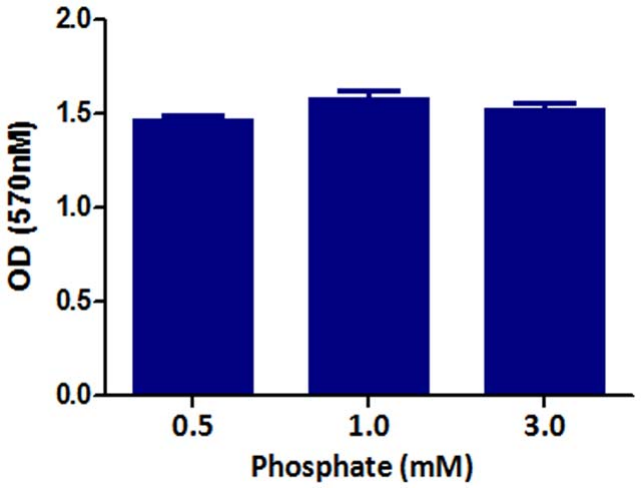
Effect of inorganic phosphate on normal human lung fibroblast proliferation. We examined the effect of exposure to different concentrations of inorganic phosphate for 24 hours on normal human lung fibroblast proliferation. The cell proliferation/viability were validated by the mitochondria-dependent reduction of MTT [3-(4,5-dimethylthiazol-2-yl)-2, 5-diphenyl tetrazolium bromide] to purple formazan as detailed in the Method section. Incubation in media simulating hypophosphatemia (0.5 mM) or hyperphosphatemia (3 mM phosphate) had no effect on fibroblast survival when compared to cells incubated in the medium containing 1 mM phosphate.

To evaluate endothelial function, we examined the effect of exposure to different concentrations of inorganic phosphate for 24 hours on endothelial cell proliferation, apoptosis, eNOS expression, and NO production. Incubation in media simulating both hypophosphatemia (0.5 mM) and hyperphosphatemia (3 mM phosphate) resulted in a significant reduction of cell numbers when compared to cells incubated in the medium containing 1 mM phosphate (Figure 1A). While the viable cells were reduced significantly in both simulated hypo- and hyperphosphatemic conditions when compared with those in normal phosphate level (Fig 1B), the necrotic cells were significantly increased only in hyperphosphatemia (Figure 1C). The cell proliferation/viability data were confirmed by the MTT assay (data not shown). Similarly, incubation in media simulating both hypophosphatemia (0.5 mM) and hyperphosphatemia (3 mM phosphate) resulted in a significant increase in apoptotic cells when compared to cells incubated in the medium containing 1 mM phosphate (Figure 1D–G). Incubation with pan-caspase inhibitor z-VAD resulted in significant reduction in apoptosis induced by both simulated hyperphosphatemia and hypophosphatemia (Figure 2 A–F). The cells incubated in media containing 0.5 mM or 3 mM phosphate showed significant reduction in eNOS abundance (Figure 3A) accompanied by parallel reductions in NO production (Figure 3B). The down-regulation of eNOS following exposure to simulated hyperphosphatemia (3 mM phosphate) was reversed with co-administration of 1 mM PFA, which is a specific inhibitor of phosphate transport across the cell membrane [26] (Figure 3C). Incubation in medium with 1 mM phosphate resulted in a significant NO production 5 minute post acetylcholine stimulation and remained elevated at 15 minutes. In contrast, incubation in media simulating both hypophosphatemia (0.5 mM) and hyperphosphatemia (3 mM phosphate) completely prevented acetylcholine-induced stimulation of NO generation, pointing to endothelial dysfunction (Fig 3D). Interestingly, simulated hyperphosphatemia and hypophosphatemia showed no effects on survival in normal human lung fibroblasts (Fig 4).

### Targeted proteomics analysis data

**Figure 5:**
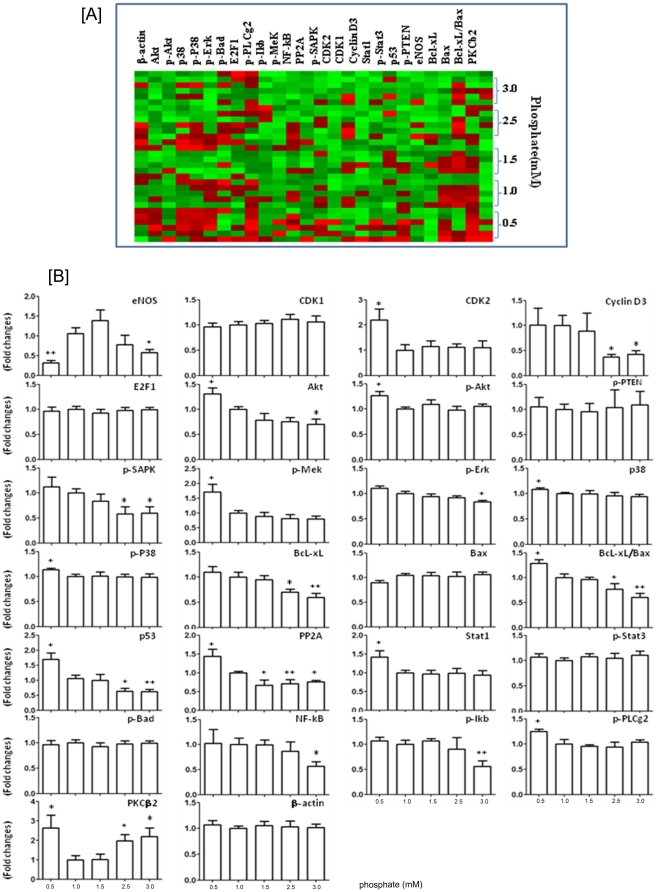
Effect of inorganic phosphate on cell signaling protein expression. Using reverse-phase protein microarray, we examined the effect of exposure to different concentrations of inorganic phosphate for 24 hours on human umbilical vein endothelial cell signaling protein expression. (A): shows the reverse-phase protein microarray heat map of key molecules for signaling pathway (green represents expression below median and red represents above-median expression). (B): shows the quantitative signaling protein expression in the endothelial cells incubated for 24 hour with different phosphate concentrations from 0.5 mM to 3.0 mM. Compared to the cells incubated with physiologic phosphate level (1.0 mM), those exposed to simulated hyperphosphatemia (3 mM) showed significantly reduced eNOS, cyclin D3, Akt, p-SAPK, p-Erk, p53, PP2A, NF-kB-p65, p-IkB, Bcl-xl , and Bcl-xl/Bax ratio, but significantly increased level of PKCβ2. On the other hand, the cells exposed to simulated hypophosphatemia (0.5 mM) showed significantly decreased level of eNOS, but significantly increased CDK2, Akt, p-Akt, p-Mek, p38, p-p38, p53, PP2A, Stat1, p-PLCg2, PKCβ2, and Bcl-xl/Bax ratio. *p<0.05, **p<0.01 vs control (1 mM phosphate).

To explore the mechanism of endothelial dysfunction induced by non-physiologic levels of inorganic phosphate, we screened the cells for levels of selected phosphorylated signaling proteins using reverse-phase protein microarray. Figure 5A shows the reverse-phase protein microarray heat map of key molecules in various signaling pathways. Figure 5B shows the quantitative signaling protein expression in the endothelial cells incubated for 24 hour with different phosphate concentrations from 0.5 mM to 3.0 mM. Compared to the cells incubated with physiologic phosphate level (1.0 mM), those exposed to simulated hyperphosphatemia (3 mM) showed significantly reduced levels of eNOS, cyclin D3, Akt, p-SAPK, p-Erk, p53, PP2A, NF-kB-p65, p-IkB, and Bcl-xl (with decreased Bcl-xl/Bax ratio), but significantly increased level of PKCβ2. On the other hand, the cells exposed to simulated hypophosphatemia (0.5 mM) showed significantly decreased level of eNOS, but significantly increased levels of CDK2, Akt, p-Akt, p-Mek, p38, p-p38, p53, PP2A, Stat1, p-PLCg2, and PKCβ2, and increased Bcl-xl/Bax ratio.

**Figure 6:**
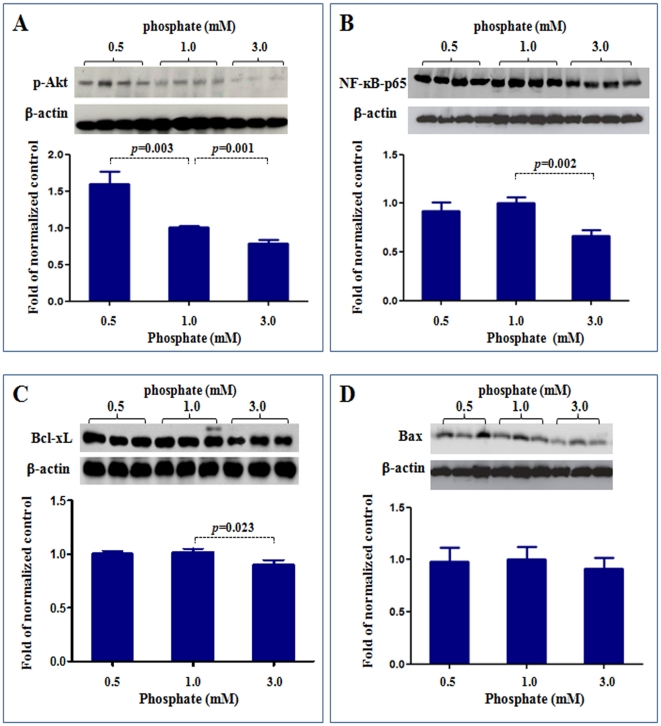
Western blot analysis of p-Akt, NFkB, Bcl-xL and Bax expressions. To validate the reverse-phase protein microarray data, we examined the protein expression for p-Akt, NF-kB-p65, Bcl-xL and Bax using western blot. Compared to the control cells exposed to physiologic phosphate level (1.0 mM), the cells exposed to simulated hypophospatemia (0.5 mM) showed significantly increased p-Akt (A), while the cells exposed to simulated hyperphospatemia (3 mM) showed significantly reduced p-Akt (A), NF-kB-p65 (B) and Bcl-xL (C). The levels of Bax were not significantly different between the three groups (D). P values vs control (1 mM phosphate). N = 4∼8.

To validate the reverse-phase protein microarray data, we examined the protein expression for p-Akt, NF-kB-p65, Bcl-xL and Bax using routine Western blot. As shown in Figure 6, Western blotting confirmed the results of reverse-phase protein microarray. Compared to the control cells exposed to physiologic phosphate level (1.0 mM), the cells exposed to simulated hypophospatemia (0.5 mM) showed significantly increased p-Akt, while the cells exposed to simulated hyperphospatemia (3 mM) showed significantly reduced p-Akt, NF-kB-p65 and Bcl-xL. The levels of Bax were not significantly different between the three groups.

### Role of Akt alteration in endothelial dysfunction

**Figure 7:**
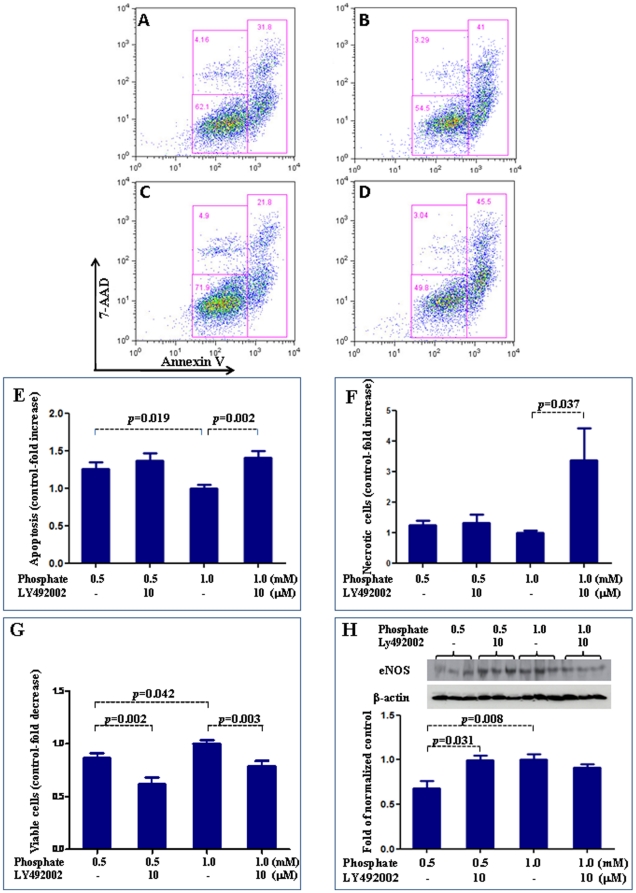
Effect of Akt pathway on endothelial function. We used PI3K/Akt inhibitor, Ly492002, to explore the role of altered Akt in the endothelial dysfunction induced by simulated hypophosphatemia. Ly492002 had no effect on increased apoptosis induced by low phosphate levels (Figure 5A–E). LY492002 resulted in decreased cell viability in hypophosphatemia. It led to decreased cell viability and increased cell death in physiologic phosphate levels (Figure 5 F & G). On the other hand, Ly492002 was able to block the reduction of eNOS expression induced by simulated hypophospatemia but had no effect on eNOS expression in cells exposed to physiologic phosphate levels (Figure 5H). In Figure A–D, data in each quadrant are the percentage of total cells, and shown as mean +/− SEM. A: 0.5 mM phosphate; B: 1 mM phosphatre; C: 0.5 mM phosphate+Ly492002; D: 1.0 mM phosphate+Ly492002.

PI3K/Akt inhibitor, Ly492002 [27], was used to explore the role of altered Akt in the endothelial dysfunction induced by simulated hypophosphatemia. Ly492002 had no effect on increased apoptosis induced by low phosphate levels (Figure 7 A–E). LY492002 resulted in decreased cell viability in hypophosphatemia. It led to decreased cell viability and increased cell necrosis in physiologic phosphate levels (Figure 7 F & G). On the other hand, Ly492002 was able to block the reduction of eNOS expression induced by simulated hypophospatemia but had no effect on eNOS expression in cells exposed to physiologic phosphate levels (Figure 7H).

### Intracellular calcium

**Figure 8:**
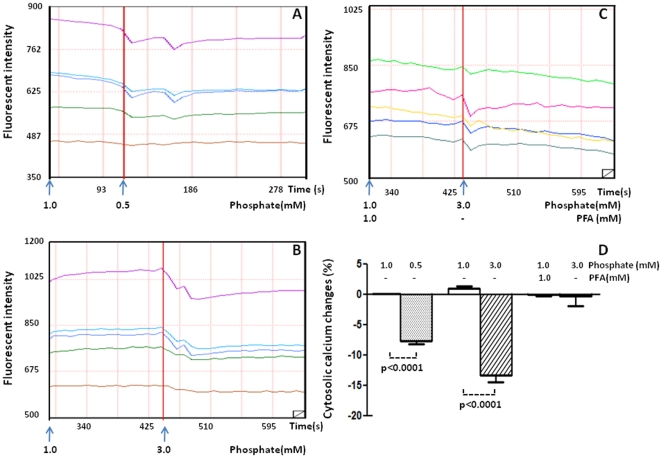
Effect of phosphate on intracellular calcium. The changes of fluorescence intensity was recorded in endothelial cells treated with 0.5 mM phosphate (A), 1 mM phosphate (B) and PFA +3 mM phosphate (C). Percentage changes in intracellular fluorescent intensity is illustrated in D. N = 5 per condition.

To study the underlying mechanism of endothelial injury induced by non-physiologic phosphate levels, we studied the effect of phosphate levels on intracellular free calcium in live cells. Significant percentage changes in intracellular calcium fluorescent intensity were observed, respectively, in cells exposed to simulated hypophosphatemia (−7.6±0.58 *vs.* 0.04±0.039, n = 5) and in cells exposed to simulated hyperphosphatemia (−13.3±1.18 *vs.* 1.02±0.31, n = 5) when compared to the normal saline control. The decrease in intracellular calcium fluorescent intensity in cells exposed to simulated hyperphosphatemia was almost completely blocked when the cells were pre-incubated with PFA, a specific inhibitor of phosphate transport across the cell membrane (−0.344±1.601 *vs.* −0.014±0.320. n = 7) (Figure 8).

## Discussion

Both hypophosphatemia and hyperphosphatemia are associated with adverse cardiovascular events. However, the underlying molecular mechanisms of these complications are not fully understood. Endothelial dysfunction is a common and critical step in the development of cardiovascular diseases and is characterized by reduced eNOS activity and/or expression and decreased NO availability, which is typical of patients with cardiovascular disease [17]. In this study, we show that exposure to both subphysiologic and supraphysiologic concentrations of phosphate reduce eNOS expression and NO production in HUVECs *in vitro*. The simulated hyperphosphatemia-induced down-regulation of eNOS expression could be reversed by co-treatment with PFA, which is a specific inhibitor of phosphate transport across the cell membrane. This indicates that the decreased eNOS expression in HUVECs exposed to simulated hyperphosphatemia is mediated by an abnormal rise in intracellular phosphate. This is consistent with the results reported by Shuto et al [10] who showed that high phosphate loading decreased NO production in brachial artery endothelial cells. They further showed that high phosphate loading activated protein kinase C (PKC), which inhibited the activity of eNOS by phosphorylating the Thr-495 residue of eNOS [10], [28]. Consistent with this previously reported result, we found that simulated hyperphosphatemia significantly increased the level of PKCβ2. In addition, we demonstrate that simulated hyperphosphatemia significantly reduced Akt abundance in the endothelial cells. The stimulatory phosphorylation of eNOS residues Ser-1177 and Ser-617 occur in response to flow shear stress that activates phosphatidyl-inositol 3-kinase (PI3K) and its downstream serine/threonine protein kinase Akt, which in turn phosphorylates eNOS (PI3K-Akt-eNOS pathway) [16], [21], [22]. Given the critical role of PI3K-Akt signaling in eNOS activation, the observed down-regulation of Akt can further diminish endothelial NO production in a hyperphosphatemic state. Further, it is known that increased intracellular phosphate levels lead to decreased calcium release from sarcomplasmic reticulum in muscle cells [29], [30]. A similar mechanism operates in endothelial cells as shown by our data of reduced intracellular free calcium in the presence of simulated hyperphosphatemia. Intracellular calcium level and calmodulin binding are critical determinants of eNOS activity [31], [32]. In addition, we observed decreased NF-kB-p65 and p-Erk abundance in the presence of simulated hyperphosphatemia. It is known that the transcription factor NF-kB initiates eNOS transcription. In the endothelial cells, the activation of NF-kB may occur with sheer stress via activation of a MAPK pathway that includes Erk1/2 or through the HDL and estrogen membrane receptor mediated PI3K-Akt pathway [23], [24], [25]. Therefore, hyperphosphatemia may reduce eNOS activity and NO production via multiple mechanisms. A limitation of our *in vitro* study is that it cannot take into account the effect of endocrine and paracrine factors such as FGF23, PTH, vitamin D, and calcium on phosphate homeostasis and endothelial function *in vitro*. Shuto et al studied the effects of hyperphosphatemia both *in vitro* and *in vitro* and showed that oxidative stress and decreased NO production in endothelial cells are possible mechanisms for the impaired endothelial function mediated by phosphate loading [10]. We did not study the production of reactive oxygen species in our experimental system.

Our study is the first to demonstrate that subnormal levels of phosphate reduce eNOS expression and NO production in HUVECs. In this regard, the effect of simulated hypophosphatemia on eNOS expression and NO production in HUVECs are similar to those seen with simulated hyperphosphatemia. Also similar to simulated hyperphosphatemia, simulated hypophosphatemia reduced intracellular free calcium and increased PKCβ2 level in HUVECs. As discussed earlier, both reduced intracellular free calcium and increased PKC would be expected to reduce eNOS activity and therefore NO production. However, surprisingly, simulated hypophosphatemia increased Akt and p-Akt levels in the endothelial cells. Similarly, we found that simulated hypophosphatemia increased the levels of p-Mek, p38, and p-p38, which are components of the MAPK pathway. Therefore, it appears that, in contrast to simulated hyperphosphatemia, simulated hypophosphatemia leads to increased PI3K/Akt and MAPK signaling, which would be expected to activate NF-kB and lead NF-kB-mediated *eNOS* transcription. Interestingly, the experiment with PI3K/Akt inhibitor Ly492002 suggests that the effect of simulated hypophosphatemia on eNOS expression may be Akt dependent. Therefore, it appears that other, yet unknown, pathways are involved that lead to reduced eNOS expression in endothelial cells incubated with subphysiologic concentrations of phosphate.

In HUVECs, both simulated hyperphosphatemia and simulated hypophosphatemia reduced endothelial cell proliferation and increased endothelial cell apoptosis. The latter is confirmed by the fact that pan-caspase inhibitor z-VAD resulted in significant reduction in apoptosis in both simulated hyper- and hypo-phosphatemia. Interestingly, simulated hyper- and hypo-phosphatemia did not affect the survival of normal human lung fibroblasts. The over-expression of the anti-apoptotic Bcl-xl protein blocks the release of cytochrome c, while the pro-apoptotic Bax protein promotes cytochrome c release from the mitochondria in response to apoptotic signals. The released cytochrome c then activates the initiator caspase-9, thereby initiating the sequential activation of downstream caspases leading to apoptosis [33], [34]. Compared to the HUVECs incubated with physiologic phosphate level, those exposed to simulated hyperphosphatemia showed significantly reduced levels of Bcl-xl and decreased the ratio of the anti-apoptotic Bcl-xl to the pro-apoptotic Bax. This suggests that endothelial cell apoptosis induced by hyperphosphatemia may act through downregulation of Bcl-xl. On the other hand, the cells exposed to simulated hypophosphatemia showed significantly increased ratio of the anti-apoptotic Bcl-xl to the pro-apoptotic Bax, suggesting that the mechanism of endothelial apoptosis in the setting of hypophosphatemia is different. The cells exposed to simulated hypophosphatemia showed elevated level of p53 (and the reverse was true for the cells exposed to simulated hyperphosphatemia) indicating that the apoptosis in the presence of low phosphate levels may be driven by p53. The non-physiologic levels of phosphate also affect the cell cycle regulatory proteins such as cyclin D3 and cyclin dependent kinase 2 (CDK2). While simulated hyperphosphatemia led to a decrease in the level of cyclin D3, simulated hypophosphatemia led to an increase in the level of CDK2 in HUVECs. Pertinently, CDK2 has been shown to play a role in inducing apoptosis and acts upstream of p53 [35], [36]. A similar CDK2-p53 dependent apoptosis may occur in the presence of hypophosphatemia. Further exploration of the alterations in cell cycle machinery in the presence of altered phosphate levels are needed to fully explain the reduced proliferation of HUVECs seen in our study. The effect of simulated hyper- and hypo-phosphatemia on endothelial cell proliferation and apoptosis indeed suggest that physiologic phosphate levels are critical for intracellular homeostasis and cell viability. These effects are likely mechanistically complex and not surprising given that phosphate is an essential component of DNA and RNA and is vital for cellular metabolism as an energy source in the form of ATP. In addition, numerous functional proteins (including caspase family of cysteine proteases that mediate apoptosis) that are essential to cellular signaling and homeostasis are activated or inactivated by reversible phosphorlyation/dephosphorylation processes catalyzed by various kinases/phosphatases [37], [38].

Our study utilized the relatively new and powerful technique of reverse phase protein array to analyze the alterations of signaling proteins in HUVECs exposed to different concentrations of phosphate. Reverse phase protein arrays are designed for quantitative, multiplexed analysis of specific active (phosphorylated, cleaved) or total forms of cellular proteins using a limited amount of sample including tissues, cells, and body fluids [39]. Reverse phase proteins arrays are more sensitive than enzyme linked immunosorbent assays (ELISA) with lower limit of detection in the attogram range and the ability to detect variances of <10%. Further, reverse phase proteins arrays do not require direct labeling of sample proteins and utilization of a two-site antibody sandwich, thereby reducing experimental variability [40], [41], [42], [43].

Given the results of this study, we would like to emphasize that researchers should pay close attention to the levels of phosphate in the culture media and adjust the level, if needed, so that phosphate is in the physiologic range. Otherwise, many molecular and biochemical processes might be affected unrelated to the experiments being performed.

In summary, we show that both simulated hyperphosphatemia and hypophosphatemia decrease eNOS expression and NO production in HUVECs and are associated with endothelial cell death. Our data provides further insights on the complex relationship between altered phosphate homeostasis and endothelial cell biology.

## Methods

### Cell culture

Human umbilical vein endothelial cells (HUVECs), purchased from Bio Whittaker (San Diego, CA, USA) were cultured in EGM endothelial cell growth medium (Lonza, Walkersville, MD) in a humidified incubator set at 37°C and 5% CO2. Once a monolayer was formed, the cells were subcultured. For the experiments, cells with no more than eight passages were used. Once reaching 90–95% confluence, the cells were subcultured in 60×15 mm BD Falcon tissue culture dishes (BD Biosciences, Franklin Lakes, NJ, USA) and incubated for 48–72 hours, at which point 85–90% confluence was reached. The cells were then rinsed with fresh medium and incubated with media containing different phosphate concentrations. Normal human lung fibroblasts (NHLF, Cat# CC-2512) were cultured in medium supplied by the manufacturer (Lonza).

### Study design

The physiologic range of serum phosphate concentration is approximately 0.9–1.5 mM. The EGM medium contains 0.5 mM phosphate (Lonza, Walkersville, MD). It was supplemented with appropriate amounts of sodium phosphate buffer (1 M Na_2_HPO_4_/NaH_2_PO_4_, pH 7.4) to achieve final inorganic phosphorus concentrations of 1.0, 1.5, 2.0, and 3.0 mM, respectively. 1 mM phosphonoformic acid (PFA) (Sigma, St Louis, MO, USA), a specific inhibitor of the sodium-phosphate transporter, was used to block phosphate transport into the cells. To determine whether abnormal phosphate levels affected the ability of acetylcholine (a physiological activator of eNOS) to stimulate NO production, a set of cells was incubated with 2 µM acetylcholine and the supernatant NO levels before (baseline) and 5 minute and 15 minute post acetylcholine stimulation was measured.

### Western blot analysis

Western blotting was performed as previously described [44]. The following primary antibodies were used: anti-eNOS (1∶5000, BD Biosciences), anti-p-Akt (1∶2000, Cell Signaling, Danvers, MA, USA), anti-NF-kB-p65 (1∶5000, Abcam, Cambridge, UK), anti-Bax (1∶2000, Cell Signaling) and ant-BcL-xL (1∶2000, Cell Signaling). Briefly, the protein content of the treated HUVEC lysate was measured using the BCA assay (Pierce Biotechnology, Rockford, IL, USA) and 20 µg total protein was size-fractionated on 4–12% Tris-glycine gel (BD Biosciences) at 100 V for 2 h. After electrophoresis, the proteins were transferred to nitrocellulose membrane (Hybond-ECL, Amersham, Buckinghamshire, UK) at 350 mA for 2.5 h using the Novex transfer system (Invitrogen, Carlsbad, CA, USA). The membrane was prehybridized in 10 ml blocking buffer (10 mmol/l Tris-HCl, pH 7.5, 100 mmol/l NaCl, 0.1% Tween-20, and 10% nonfat milk powder) for 1 hour (room temperature) and then hybridized for an additional 12 hours at 4°C in the same buffer with added primary antibody. Then the membrane was washed for 30 min in a shaking bath; the wash buffer (Tris-buffered saline Tween 20) was changed every 10 min for 3 times. Then the membrane was incubated for 1 hour with block buffer plus secondary antibody tagged with horseradish peroxidase at the final titer of 1∶5000. The washes were repeated before the membrane was developed with a light emitting nonradioactive method using ECL reagent (SuperSignal West Dura kit, Thermo Scientific, Rockford, IL, USA). The membrane was then subjected to autoluminography for 5 min. The respective band intensities were measured using Scion Image (WinB403; http://rsb.info.nih.gov/index). In all instances, the membranes were stained with Ponceau stain, which verified the uniformity of protein load and transfer efficiency across the test samples.

### Apoptosis assay

Apoptotic cells were determined by staining with the Annexin V-PE Apoptosis Kit (BD, Pharmingen), which employs a FITC-labeled annexin V (Annexin V-FITC) in concert with 7-Amino-actinomycin D (7-AAD) to evaluate subpopulations of cells undergoing apoptosis. Briefly, after 24-hour incubation in the medium described above, the cells were digested with 0.05% Trypsin-EDTA (Invitrogen, Carlsbad, CA, USA) for 1 min, harvested using the medium containing 2% FBS, and collected by centrifugation (200 g) at 4°C. The cells were re-suspended at a final concentration of 2×10^7^ cells/ml. 2×10^5^ cells (100 µl) were washed with cold staining buffer (PBS with calcium and magnesium supplemented with 0.5% FCS and 0.5% NaN_3_) and then incubated with 5 µl annexin V-FITC and 5 µl 7-AAD in 100 µl staining buffer, for 15 min, at 4°C, in the dark. Cells were washed again, gently re-suspended in 250 µl staining buffer. Cell samples were analyzed by flow cytometry on a FACScan flow cytometer (Becton-Dickinson, Mountain View, CA, USA) and ten thousand events were counted. Live cells are Annexin-V-ve, 7-AAD-ve; necrotic cells are Annexin-V-ve, 7-AAD+ve; apoptotic cells are Annexin-V+ve, 7-AAD-ve (early apoptosis) or Annexin-V+ve, 7-AAD+ve (late apoptosis) [45], [46]. To separate apoptosis from necrosis, a pan-caspase inhibitor, z-VAD (40 uM; Cat# N-1510, Z-Val-Ala-DL-Asp-fluoromethylketone) [47] was added to the media with simulated hypophosphatemia (0.5 mM) or simulated hyperphosphatemia (3.0 mM) for 24 hrs and flow cytometry analyses were performed as described above.

### Cell proliferation studies

The cell number was determined by a Counting Chamber (Levy Counting Chamber, Hausser Scientific, Horsham, PA, USA). For counting the cells, cultured cell samples were digested with 0.05% trypsin-EDTA, harvested using the medium with 2% FBS, and collected by centrifugation (200×g) for 5 min at 4°C. Cells were re-suspended with 1 ml culture medium and thoroughly mixed. 100 µl of suspension cells was used to count the number of cells. In addition, cell proliferation/viability were validated by the mitochondria-dependent reduction of MTT [3-(4,5-dimethylthiazol-2-yl)-2, 5-diphenyl tetrazolium bromide] (Sigma) to purple formazan. Briefly, cells were seeded on to 96 well culture plates and treated with different concentrations of phosphate for 24 h. After the treatment, 100 ug MTT reagent was added to each well. After 4 h of incubation, the supernatant was carefully removed and 150 µl of dimethyl sulfoxide solution was added to dissolve the formazan crystals. After shaking for 10 min on a plate mini-shaker, the absorbance at 570 nm was read on a microplate reader.

### Measurement of NO

NO production was assessed by measurement of NO metabolites, nitrates plus nitrites, in the cell culture media using QuantiChrom Nitric Oxide Assay Kit (BioAssay Systems, Hayward, CA) according to the manufacturer's instructions. This assay is based on the reduction of nitrate to nitrite by cadmium and the subsequent detection of nitrite with the Griess reagent which is specific for nitrite. The detection range is 0.1–50 uM in 96-well plate.

### Reverse phase protein array

Reverse phase protein array was used to query the HUVEC lysates for signaling proteins as described previously [48], [49], [50]. Antibodies to Akt, p-AKT, p-Stat3, P38, p-P38, p-ErK1,2, p-Bad, E2F1, p-PLCr2, p-IkB, p-Mek, NF-kB-p65, p-PTEN, Bcl-xL, Bax and p-STAT3 were purchased from Cell Signaling. Antibodies to eNOS, PP2A, CDK1, CDK2, cyclin D3, Stat1, p53, and PKCβ2 were purchased from BD Biosciences. Antibody to β-actin was purchased from Epitomics. All antibodies were used at dilutions of 1∶250–1000. HUVECS cultured in media containing various concentrations of phosphate were harvested and lysed. Protein concentrations of the lysates were determined using the BCA assay (Pierce Biotechnology, Rockford, IL, USA). All lysates, each in four dilutions (neat, 1∶3, 1∶9, 1∶27, 1∶81) were arrayed on nitrocellulose coated glass slides and stored at −80°C till further analysis. The slides were blocked and then incubated with the primary antibodies. After incubating with biotinylated secondary antibody, the array slides were developed with the Dako signal amplification system using catalyzed reporter deposition of substrate (Dako, Carpinteria, CA, USA). The developed slides were scanned as TIFF files and the signals analyzed with Imagequant TL (GE Healthcare, Wauwatosa, Wisconsin, USA). β-actin was used as a loading control to normalize data.

### Measurement of intracellular free calcium

To investigate the effect of phosphate on intracellular free calcium in live cells, the fluo-3 acetoxymethyl (AM) (Fluo3-AM) ester technique was used [51]. Briefly, the cells were seeded on poly-D-lysine-coated coverslips in 2.5×2.5 cm^2^ well tissue culture and maintained in a humidified incubator at 37°C in an atmosphere of 5% CO2 in air. Once a monolayer reached 50–60% confluence, the cells were rinsed and incubated in Fluo3-AM calcium dye (Molecular Probes, Eugene, OR) for 15 min at 37°C and excess dye was removed. For confocal studies, dye-loaded endothelial cells on coverslips were incubated with culture medium with normal phosphate (1.0 mM), followed by normal saline (control) or simulated hypophosphatemia (0.5 mM) or simulated hyperphosphatemia (3.0 mM) and PFA. A baseline reading of dye-loaded cells was measured with a fluorometer at 485 nm emission/530 nm excitation (Perseptive Biosystems, Stafford, TX). The experimental reading was taken continuously, and raw data were collected for 10 second intervals. The percent changes in fluorescence were calculated and graphed using the Origin macroanalysis software. Five wells were used for each treatment, and individual responses were averaged to represent the value for a single experiment. All experiment procedures were performed in less than 5 minutes.

### Statistical Analyses

Data are presented as mean ± SEM. Analysis of variance (ANOVA) and Student's t-test were used in statistical evaluation of the data as appropriate. P-values less than or equal to 0.05 were considered significant.
